# Contraceptive implant use duration is not associated with breakthrough pregnancy among women living with HIV and using efavirenz: a retrospective, longitudinal analysis

**DOI:** 10.1002/jia2.26001

**Published:** 2022-09-08

**Authors:** Randy M. Stalter, Gustavo Amorim, A. Rain Mocello, Beatrice Jakait, Bryan E. Shepherd, Beverly Musick, Caitlin Bernard, Elizabeth A. Bukusi, Kara Wools‐Kaloustian, Craig R. Cohen, Constantin T. Yiannoutsos, Rena C. Patel, the Implant/Efavirenz Study Group and the East Africa IeDEA regional consortium

**Affiliations:** ^1^ Department of Epidemiology University of Washington Seattle Washington USA; ^2^ Department of Biostatistics Vanderbilt University Nashville Tennessee USA; ^3^ Bixby Center for Global Reproductive Health and Department of Obstetrics, Gynecology & Reproductive Sciences University of California San Francisco San Francisco California USA; ^4^ Moi Teaching & Referral Hospital/Moi University & Academic Model Providing Access to Healthcare (AMPATH) Eldoret Kenya; ^5^ Department of Biostatistics and Health Data Science School of Medicine Indiana University Indianapolis Indiana USA; ^6^ Division of Family Planning Department of Obstetrics & Gynecology Indiana University School of Medicine Indianapolis Indiana USA; ^7^ Centre for Microbiology Research Kenya Medical Research Institute Nairobi Kenya; ^8^ Department of Medicine Indiana University School of Medicine Indianapolis Indiana USA; ^9^ Department of Biostatistics and Health Data Science R.M. Fairbanks School of Public Health Indiana University Indianapolis Indiana USA; ^10^ Division of Allergy and Infectious Diseases Department of Medicine and Department of Global Health University of Washington Seattle Washington USA

**Keywords:** HIV, women living with HIV, contraception, efavirenz, implant, pregnancy

## Abstract

**Introduction:**

Contraceptive implants containing etonogestrel and levonorgestrel have emerged as popular contraceptive options among women in areas of high HIV burden in sub‐Saharan Africa. However, recent pharmacokinetic data have shown drug–drug interactions between implants and efavirenz‐containing antiretroviral therapy (ART), reducing the effectiveness of the implants. Here, we evaluated pregnancy incidence in 6‐month intervals following implant initiation among women using efavirenz and contraceptive implants to assess whether the risk of breakthrough pregnancy is higher after specific periods of implant use.

**Methods:**

We used data from a retrospective longitudinal analysis of women living with HIV ages 18–45 years in western Kenya who attended HIV‐care facilities between 2011 and 2015. We used Cox proportional hazard models to compute hazard ratios (HRs) for breakthrough pregnancy by implant type and ART regimen. Depending on the model, we adjusted for socio‐demographic and clinical factors, programme, site and interaction between calendar time and ART regimen. We utilized inverse probability weights (IPWs) to account for three sampling phases (electronic medical record [EMR], chart review and phone interview) and calculated overall parameter estimates.

**Results:**

Women contributed 14,768 woman‐years from the largest sampling phase (EMR). The median age was 31 years. Women used etonogestrel implants for 26–69% of the time and levonorgestrel implants for 7–31% of the time, depending on the sampling phase. Women used efavirenz, nevirapine or no ART for 27–33%, 40–46% and 15–26% of follow‐ups, respectively. When combining sampling phases, there was little evidence to suggest that the relative hazard of pregnancy among efavirenz‐containing ART users relative to nevirapine‐containing ART changed with length of time on implants: IPW‐adjusted HR of 3.1 (CI: [1.5; 6.4]) at 12 months, 3.4 (CI: [1.8; 6.3]) at 24 months, 3.8 (CI: [1.9; 7.7]) at 36 months and 4.2 (CI: [1.6; 11.1]) at 48 months (interaction *p*‐value = 0.88). Similarly, no significant change in HRs over time was found when comparing women not using ART to nevirapine‐containing ART users (interaction *p*‐value = 0.49).

**Conclusions:**

We did not find evidence to suggest implants being more fallible from drug–drug interactions with efavirenz at later time intervals of implant use. Thus, we would not recommend shortening the duration of implant use or replacing implants sooner when concomitantly used with efavirenz.

## 1 INTRODUCTION

In sub‐Saharan Africa, over half of pregnancies among women living with HIV (WLHIV) are unintended [1]. Unplanned pregnancy is associated with an increased risk of maternal morbidity and mortality and mother‐to‐child HIV transmission.[2, 3, 4, 5] Reducing unplanned pregnancies by use of family planning, including effective contraception, is one of the four pillars of preventing mother‐to‐child transmission of HIV [6, 7]. While injectable contraceptives are the predominant method of birth control in sub‐Saharan Africa, there has been increased use of contraceptive implants, one of the most effective contraceptive methods, over the past decade [8]. Existing subdermal implants come as single or double rods, containing the progestins etonogestrel or levonorgestrel and are formulated to last at least 3 or 5 years, respectively. In addition to their superior efficacy, implants also allow for discreet use without the need for user action, have a long duration of use, are reversible and do not interfere with sex, which makes them an ideal method for many women [9]. Ten countries in sub‐Saharan Africa now have an implant use prevalence of >5% among married women, compared to none a decade ago [10]. Implant use is particularly high in areas with high HIV prevalence. In Kenya, specifically, contraceptive implant use increased from 2% in 2008 to 18% in 2016 among married women [8].

While these trends in implant use are encouraging, studies conducted among WLHIV have revealed drug–drug interactions between contraceptive implants and the previously recommended first‐line antiretroviral medication, efavirenz, due to induction of CYP450 enzymes. Pharmacokinetic studies have demonstrated 40–82% reductions in serum hormone concentrations in women concomitantly using implants with efavirenz [11, 12, 13, 14]. Clinical and epidemiological studies have linked combined implant and efavirenz use with higher pregnancy risk [15, 16]. The largest study to date among over 80,000 WLHIV between the ages of 15–45 years in Kenya showed a three‐fold higher risk of incident pregnancy among women using implants with efavirenz‐based antiretroviral therapy (ART) compared to women using implants with nevirapine‐based ART [17].

With continuous product use, systemic implant hormone concentrations gradually decrease over time, with concentrations highest within the first 2 weeks after implant insertion and then with a somewhat logarithmic decline over the first year to, finally, a long, steady “tail” after that first year of use [18, 19]. Whether contraceptive implant use may be more fallible to drug–drug interactions that may reduce effectiveness after specific periods of use due to these decreases in hormone concentrations is unknown. Here, we assess pregnancy incidence within 6‐month intervals following implant initiation among WLHIV using efavirenz and contraceptive implants.

## 2 METHODS

### 2.1 Study setting and population

For this secondary analysis, we leveraged data previously collected for a retrospective longitudinal analysis to assess pregnancy risk among women concomitantly using ART and implants [17, 20, 21]. Data were collected from WLHIV ages 18–45 years in western Kenya who attended HIV‐care facilities between 1 January 2011 and 25 December 2015. All facilities were supported by the Academic Model Providing Access to Healthcare (AMPATH) or Family AIDS Care & Education Services (FACES), two PEPFAR‐supported programmes affiliated with the East Africa International Epidemiology Databases to Evaluate AIDS (EA‐IeDEA) consortium [22]. AMPATH and FACES collectively support approximately 130 facilities and provide comprehensive HIV care and treatment services to nearly 200,000 people living with HIV, including in Bungoma, Busia, Elgeyo Marakwet, Kisumu, Nandi, Tans Nzoi, Uashin Gishu and West Pokot for AMPATH and Kisumu, Homay Bay and Migori counties for FACES. Clients attending these facilities have access to family planning services and safe conception counselling at no or low out‐of‐pocket costs. The primary study included all WLHIV, regardless of contraceptive method type used, and utilized a three‐phase sampling strategy (the first phase used electronic medical records [EMRs], the second phase employed manual chart review and the third phase conducted telephone interviews with clients) to validate the estimates generated from the EMR analysis. The manual chart reviews and phone interviews occurred with sequential subsets of clients and were conducted from April 2016 to March 2017.

For this analysis, we included all women who had at least one clinic visit with a report of any type of contraceptive implant use in the EMR or chart review or had reported implant use during their phone interview during the study observation period. Thus, though the chart review and phone interviews occurred sequentially, women included in this analysis are not necessarily a subset of the prior sampling phase, as women could have a record or self‐report of implant use not captured in the prior sampling phase.

The Human Subjects Division at the University of Washington, Indiana University Institutional Review Board, Committee on Human Research at the University of California, San Francisco, Institutional Research and Ethics Committee at Moi University, Ethical Review Committee at Kenya Medical Research Institute and U.S. Centers for Disease Control and Prevention approved this research. This analysis conforms to the ethical standards established by the Declaration of Helsinki. Individual informed oral consent was obtained from participants undergoing the telephone interviews.

### 2.2 Measures

#### 2.2.1 Outcome

Our primary outcome was a documented, new pregnancy that was either confirmed via clinical diagnosis (i.e. the woman presenting gravid) or based on self‐reports. In this study setting, urine/serum tests were not routinely conducted to screen for or verify probable pregnancies nor prior to contraceptive initiation. We estimated the date of incident pregnancy using clients’ likely date of conception, which we based on self‐reports of last menstrual period, estimated gestational age or estimated date of delivery. We assumed that if an implant was not explicitly reported as having been removed or another contraceptive method had not been initiated prior to a pregnancy detection, the implant was still being utilized. We followed women for 9 months after December 2015 to detect any pregnancies that may have been conceived at the end of our study period but were not yet clinically identified.

#### 2.2.2 Exposures

Information on women's exposures to contraceptive methods and ART was initially collected from the clinic EMR. From each clinic visit, we abstracted information on the type of contraceptive implant women were currently using and classified the implant into one of the following categories: (1) levonorgestrel implant, (2) etonogestrel implant or (3) unknown implant type. If the records indicated that an implant was used concomitantly with another contraceptive method, we assigned the contraceptive method type as the implant as long as the method did not have higher effectiveness (i.e. intrauterine devices or surgical methods).

Regarding ART regimen exposure, we classified women as being on either efavirenz‐containing ART, nevirapine‐containing ART or no ART. Due to few observations among women using protease inhibitor (PI)‐containing ART, ART containing only nucleos(t)ide reverse transcriptase inhibitors (NRTIs) or combination regimens (i.e. combinations of antiretrovirals that include more than one non‐NRTI class of drugs, such as containing NRTIs and efavirenz and PIs), observations with these categories were excluded from the analysis. We chose the use of nevirapine‐containing ART as the reference category for ART comparisons across contraceptive methods, as the alternative option of no ART is not clinically meaningful in the era of universal ART use.

#### 2.2.3 Covariates

The number of living children, marital status and education level were documented at enrolment in care, though marital status was recorded at multiple visits at AMPATH. Age, CD4 cell count, WHO Clinical Stage, use of tuberculosis medications and calendar time were also collected multiple times, with average age calculated for each observation period (i.e. period of pregnancy risk with unchanged ART and implant type), CD4 cell count and WHO Clinical Stage documented closest to the start of each period, and use of tuberculosis medications documented at any point during the period. We used the documented body weight value closest to the start of the observation period and height during enrolment in care or closest to the start of the observation period. Body mass index was calculated using weight in kilograms divided by height in metres [2] at the start of each observation period and considered time‐varying. If weight was <30 kg or height was <100 cm, we replaced the value with a backward and then forward imputation, when values temporally preceding or following the visit which contained the missing value were available, respectively. If such adjacent values were not available, we used multiple imputations by chained equations (MICE) to replace these unreliable values. A more detailed explanation of these factors may be found elsewhere [17, 20, 21].

### 2.3 Statistical analysis

Categorical variables were presented as frequencies and proportions, whereas continuous variables were presented using medians and interquartile range. Data that were missing in the EMR were imputed via MICE. The imputation model used contraceptive methods, ART regimens, pregnancy status, as well as all other covariates used in the regression analysis. Details on the imputation models are described elsewhere [17].

Like the parent study, for this analysis, we utilized the three sampling phases to overcome potential limitations in data collection and entry errors in the EMR [17]. The hazard for a breakthrough pregnancy for each implant type was first estimated via Cox proportional hazards model for each dataset (EMR, chart review and telephone interview) individually. The effect of the exposure variable (ART regimen) was allowed to vary linearly on time by including an ART‐by‐time interaction in the hazard function of the Cox model, relaxing the proportional hazards assumption. Due to limited sample size, the analysis using the chart review and telephone interview datasets adjusted for age and programme (AMPATH or FACES) only, while analysis using the EMR dataset adjusted, in addition, for body mass index (log‐scale) and an indicator variable of whether the woman had at least one living child. Kaplan–Meier curves, presenting the proportion of women who did not have a breakthrough pregnancy (“survival” outcome), were also generated for each dataset separately.

A Cox proportional hazard model with inverse probability weighting (IPW) was used to compute adjusted hazard ratios (HRs) for breakthrough pregnancy for each implant type, using all three datasets (EMR, chart review and telephone interview) in the same analysis. This IPW approach works by weighting each fully validated woman (i.e. women who were selected for collection of EMR, chart review and telephone interview data) by the inverse of her probability of being selected for the telephone interview. More specifically, if we define *p_1_* and *p_2_* as the probabilities of being selected for the chart review and subsequently for the telephone interview, respectively, the weights are calculated as 1*/(p_1_p_2_)*. This weighted approach estimates the quantity that would have been obtained had all records been fully validated and accounts for the fact that pregnant women from specific combinations of ART regimens and contraceptive types were more likely to be sampled for validation. Confidence intervals were estimated using robust standard errors. A complete case analysis using unimputed datasets that excluded all observations with missing covariate values was carried out to assess for bias associated with covariates potentially not being missing at random.

We prepared the data using SAS version 9.4 (SAS Institute, Cary, NC, USA) and conducted analyses using R version 4.0.5 (R Core Team, Vienna, Austria).

## 3 RESULTS

### 3.1 Baseline characteristics and distribution of implant type and ART regimen

Women included in this analysis contributed 14,768 woman‐years (14,295 women) from the EMR dataset, 6604 woman‐years (2976 women) from the chart review dataset and 2102 woman‐years (750 women) from the telephone interview dataset (Table 1). Women contributed a median of 0.4, 2.3 and 2.0 women‐years of follow‐up within these three sample phases, respectively. In the largest of the sampling phases, the EMR phase, women had a median age of 31 years. Women were married for 59% of the total observation time, mostly had some primary education (40% of the time) and had at least one living child for 69% of the time. Regarding clinical characteristics, women had a WHO Clinical Stage of 1 for 44% of the time, had a median body mass index of 22 and were on active tuberculosis treatment for 6% of the time.

**Table 1:** General characteristics of women sampled in each phase, based on woman‐years contributed to each sample phase, 1 January 2011–31 December 2015

Characteristics^a^	EMR (First phase)	Chart review (Second phase)	Telephone interview (Third phase)
Total women years (total number of women)	14,768 (14,295)	6604 (2976)	2102 (750)
Number of observations per woman, median (IQR)	1 (1–1)	1 (1–1)	1 (1–1)
Total observation time per woman in years, median (IQR)	0.4 (0.1–1.2)	2.3 (1.2–3.2)	2.0 (0.8–3.3)
Age at the start of the observation period, median (IQR)	31 (26–36)	31 (27–35)	30 (26–34)
Implant type			
Etonogestrel	6805 (46%)	1727 (26%)	1440 (69%)
Levonorgestrel	1139 (8%)	1510 (23%)	649 (31%)
Unknown type	6589 (45%)	3367 (51%)	13 (1%)
Missing	235 (2%)	0 (0%)	0 (0%)
ART regimen			
Efavirenz‐containing	4046 (27%)	2168 (33%)	582 (28%)
Nevirapine‐containing	6779 (46%)	2991 (45%)	837 (40%)
PI‐containing	945 (6%)	477 (7%)	134 (6%)
No ART	2747 (19%)	968 (15%)	549 (26%)
Missing	250 (2%)	0 (0%)	0 (0%)
Education level			
Completed college	26 (0%)	23 (0%)	4 (0%)
Some college/university	298 (2%)	157 (2%)	63 (3%)
Completed secondary	629 (4%)	364 (6%)	80 (4%)
Some secondary	1703 (12%)	739 (11%)	191 (9%)
Completed primary	1686 (11%)	947 (14%)	166 (8%)
Some primary	5925 (40%)	2561 (39%)	484 (23%)
None	48 (0%)	2 (0%)	0 (0%)
Missing	4452 (30%)	1812 (27%)	1113 (53%)
Marital status			
Legally married	8748 (59%)	4122 (62%)	1197 (57%)
Living w/partner	102 (1%)	79 (1%)	33 (2%)
Never married and not living w/partner	704 (5%)	277 (4%)	65 (3%)
Separated/divorced	1307 (9%)	594 (9%)	252 (12%)
Widowed	1645 (11%)	737 (11%)	191 (9%)
Missing	2261 (15%)	794 (12%)	364 (17%)
Number of living children			
0	941 (6%)	557 (8%)	628 (30%)
1+	10,118 (69%)	4471 (68%)	1474 (70%)
Missing	3709 (25%)	1576 (24%)	0 (0%)
WHO Clinical Stage			
1	6526 (44%)	3093 (47%)	968 (46%)
2	4458 (30%)	1848 (28%)	608 (29%)
3	3123 (21%)	1388 (21%)	455 (22%)
4	574 (4%)	271 (4%)	70 (3%)
Missing	88 (1%)	5 (0%)	0 (0%)
CD4 cell count (cells/μl)			
Median (IQR)	481 (327–670)	490 (345–680)	481 (325–670)
Weight (kg)			
Median (IQR)	57 (52–64)	58 (52–65)	59 (53–66)
Bo (kg/m^2^)			
Median (IQR)	22 (20–24)	22 (20–24)	22 (20–25)
Active tuberculosis treatment			
None	13,949 (94%)	6140 (93%)	1949 (93%)
Active treatment	819 (6%)	463 (7%)	153 (7%)
Programme			
AMPATH	8428 (57%)	3114 (47%)	1343 (64%)
FACES	6340 (43%)	3490 (53%)	759 (36%)
Calendar year			
2011	1162 (8%)	1573 (24%)	822 (39%)
2012	3261 (22%)	2607 (39%)	590 (28%)
2013	3679 (25%)	1394 (21%)	376 (18%)
2014	4651 (32%)	800 (12%)	196 (9%)
2015	2014 (14%)	230 (3%)	117 (6%)

Abbreviations: ART, antiretroviral therapy; EMR, electronic medical record; IQR, interquartile range; PI, protease inhibitor; WHO, World Health Organization.

^a^ ART regimen and implant type are considered time‐varying exposures. All other variables are ascertained at the start of the ART/contraceptive combination category and assumed constant within an ART/contraceptive combination category but allowed to vary between ART/contraceptive combination categories.

The proportion of time women were using each type of implant varied by sampling phase, ranging from 26% to 69% of the time using the etonogestrel implant and 7% to 31% of the time using the levonorgestrel implant. In terms of ART type, efavirenz was used for 27–33% of follow‐ups, nevirapine was used for 40–46% of follow‐ups and no ART was used for 15–26% of follow‐ups, depending on the sampling phase.

### 3.2 Cumulative incidence and instantaneous hazard of breakthrough pregnancy are stable over time

**Figure 1:**
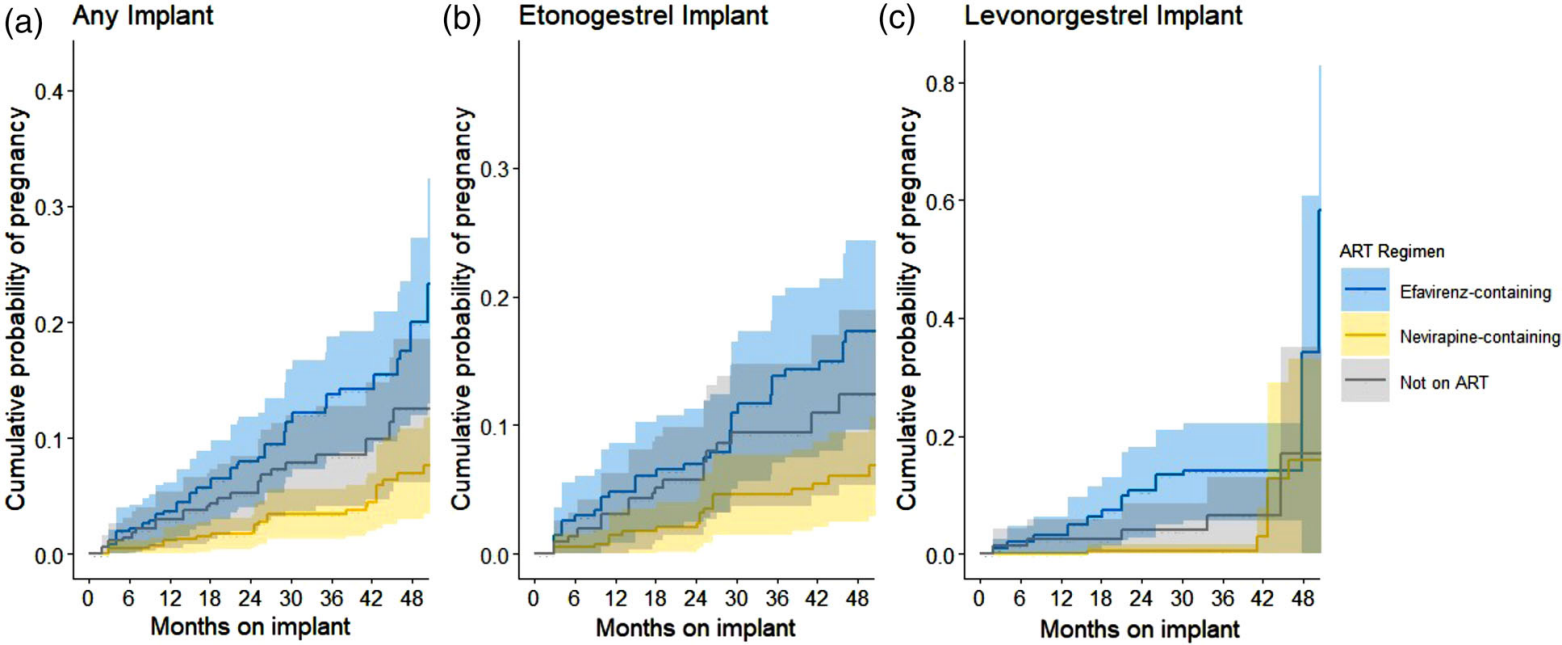
Weighted Kaplan–Meier curves of cumulative probability of breakthrough pregnancy among women using contraceptive implants, by implant type and ART regimen. (a) Any implant; (b) etonogestrel implant; and (c) levonorgestrel implant. Women's contraceptive and antiretroviral therapy exposures were validated in this study using three separate data phases: electronic medical record abstraction, chart review and telephone interview. Each woman fully validated using these three data phases was weighted by the inverse of her probability of being selected for the telephone interview. The solid lines in each curve indicate the estimated weighted cumulative pregnancy probabilities for that particular ART regimen each month for women using the respective implant. The shaded areas surrounding each line indicate the 95% confidence interval.

**Figure 2:**
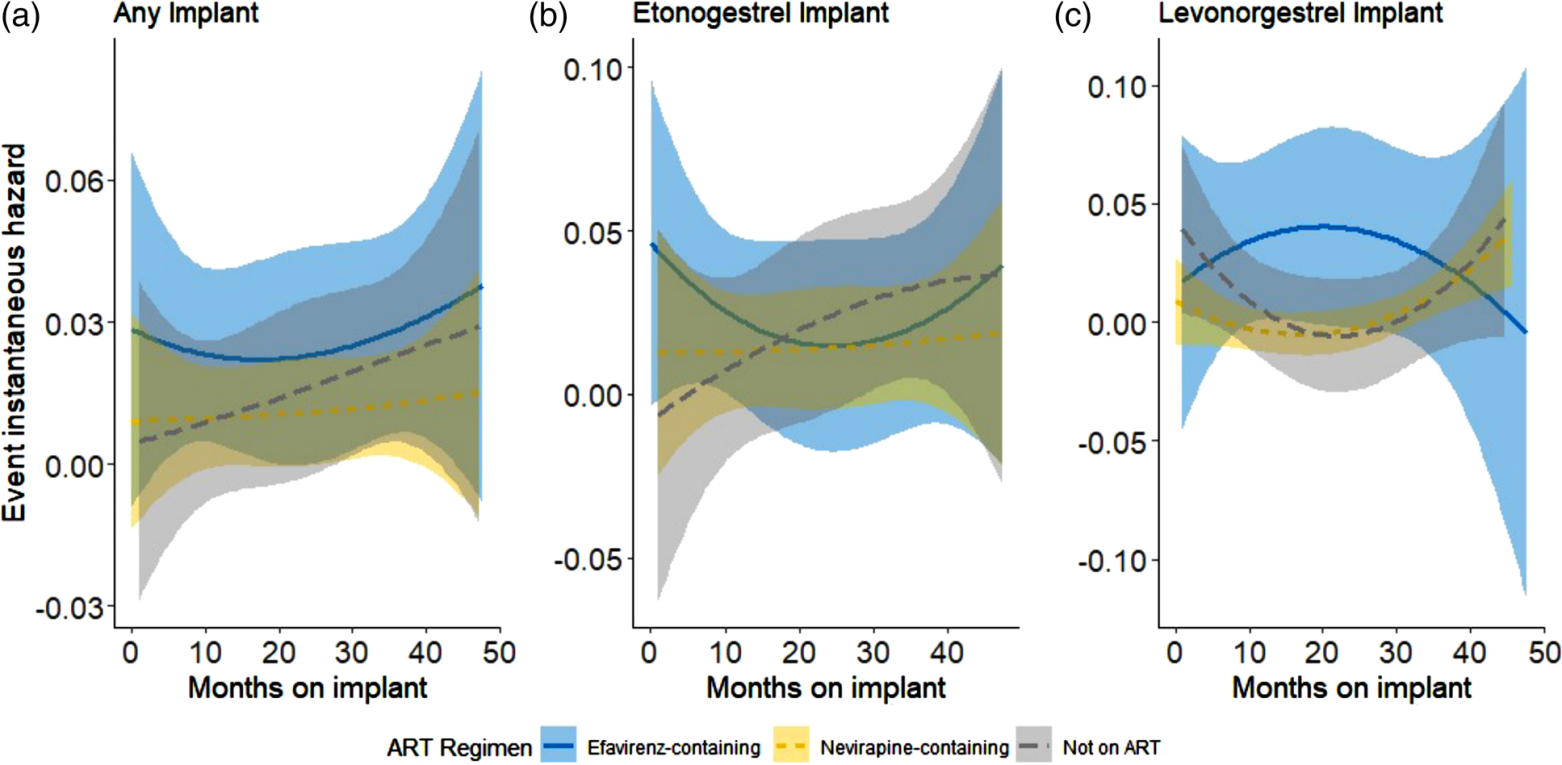
Weighted instantaneous hazard of breakthrough pregnancy among all sampled women, by implant type. (a) Any implant; (b) etonogestrel implant; and (c) levonorgestrel implant. Women's contraceptive and antiretroviral therapy exposures were validated in this study using three separate data phases: electronic medical record abstraction, chart review and telephone interview. Each woman fully validated using these three data phases was weighted by the inverse of her probability of being selected for the telephone interview. Curves indicate the estimated weighted instantaneous hazard of breakthrough pregnancy for that particular ART regimen each month for women using the respective implant. Each smoothed curve was fitted via a restricted cubic spline with three knots equally spaced. The shaded areas surrounding each smoothed curve indicate the 95% confidence interval.

Among the cohort of implant users selected for a telephone interview, which was the cohort included in the combined Cox regression models, there were 19 incident pregnancies documented across 837 woman‐years among nevirapine users, 42 incident pregnancies across 582 woman‐years among efavirenz users and 22 incident pregnancies across 549 woman‐years among women not using ART. The estimated number of pregnancies and woman‐years expected to be observed among women in the full EMR cohort after weighting each fully validated woman by her inverse probability of being selected for the telephone interview are displayed in Table 2. Figures 1 and 2 illustrate the weighted cumulative incidence and instantaneous hazard of breakthrough pregnancy across times of implant use by ART and implant type, respectively. Stratification of the instantaneous hazards by sampling type can be found in Figure S1. The instantaneous hazard curves indicate no clear inflection point whereby there are increased and sustained rates of breakthrough pregnancy after a certain period of implant use.

**Table 2:** Risk of breakthrough pregnancy over time among women using contraceptive implants and nevirapine‐containing versus efavirenz‐containing ART, 1 January 2011–31 December 2015

	Nevirapine users		Efavirenz users		Not using ART		Efavirenz versus nevirapine	No ART versus nevirapine
Months since implant insertion	Women at risk^a^	Pregnancies^a^	Women at risk^a^	Pregnancies^a^	Women at risk^a^	Pregnancies^a^	IPW^b^aHR (95% CI)	IPW^b^aHR (95% CI)
Any implant^c^								
6 months	3995	20	2497	60	2626	40	2.9 (1.2–7.0)	1.8 (0.6–5.2)
12 months	3739	24	2219	36	2305	38	3.1 (1.5–6.4)	1.9 (0.8–4.6)
18 months	3403	12	2011	45	1981	24	3.2 (1.7–6.2)	2.0 (1.0–4.3)
24 months	3061	8	1868	46	1689	23	3.4 (1.8–6.3)	2.2 (1.1–4.3)
30 months	2551	52	1743	67	1301	44	3.6 (1.9–6.8)	2.3 (1.1–4.7)
36 months	2069	0	1292	39	1110	8	3.8 (1.9–7.7)	2.4 (1.1–5.5)
42 months	1482	18	1054	6	705	11	4.0 (1.7–9.1)	2.6 (1.0–6.7)
48 months	979	31	549	49	524	17	4.2 (1.6–11.1)	2.7 (0.9–8.5)
Etonogestrel implant								
6 months	2556	14	1420	46	1734	24	2.2 (0.8–6.6)	1.5 (0.5–4.9)
12 months	2461	24	1219	25	1556	30	2.5 (1.0–6.1)	1.7 (0.6–4.5)
18 months	2300	6	1208	16	1352	24	2.7 (1.2–5.9)	1.8 (0.8–4.3)
24 months	2068	8	1161	12	1201	14	3.0 (1.5–6.1)	2.0 (0.9–4.4)
30 months	1755	52	1087	50	971	44	3.3 (1.6–6.7)	2.1 (0.9–4.9)
36 months	1573	0	969	33	846	0	3.6 (1.6–7.9)	2.3 (0.9–6.0)
42 months	1236	12	843	6	583	11	4.0 (1.6–9.9)	2.5 (0.8–7.7)
48 months	820	6	483	22	448	8	4.3 (1.5–12.9)	2.7 (0.7–10.3)
Levonorgestrel implant								
6 months	1393	0	1083	24	892	16	14.7 (3.1–68.8)	5.5 (0.6–47.0)
12 months	1232	0	995	12	749	8	12.2 (3.1–48.2)	4.9 (0.8–31.0)
18 months	1075	6	799	29	622	0	10.1 (2.8–36.1)	4.5 (0.9–21.6)
24 months	976	0	685	35	489	9	8.4 (2.4–29.5)	4.0 (1.0–16.2)
30 months	778	0	594	17	331	0	6.9 (1.8–26.3)	3.6 (1.0–13.6)
36 months	496	0	280	6	264	8	5.8 (1.3–25.3)	3.3 (0.8–13.1)
42 months	246	6	156	0	122	0	4.8 (0.9–25.7)	3.0 (0.6–14.0)
48 months	158	24	55	17	76	9	4.0 (0.6–27.1)	2.7 (0.4–16.4)

Abbreviations: aHR, adjusted hazard ratio; ART, antiretroviral therapy; CI, confidence interval; IPW, inverse probability weighting.

^a^ Values obtained after weighting patients in the telephone interview dataset by the inverse of their probability of being selected for full validation; values are rounded.

^b^ Cox regression model with inverse probability weighting, adjusting for ART regimen and its interaction with time, age and programme (AMPATH or FACES), with robust standard errors.

^c^ Includes women using etonogestrel implants, levonorgestrel implants or implants of unknown type. Weighted hazard ratios could not be estimated in the unknown implant group due to small sample size in the phone interview dataset; therefore, results from this subgroup have been excluded. Estimates for the unknown implant group for the individual sampling phases can be found in Table S1.

Among all included women with any implant, IPW‐adjusted HRs of pregnancy at 12, 24, 36 and 48 months of implant use for efavirenz‐containing ART users were somewhat consistent over time at 3.1 (CI: [1.5; 6.4]), 3.4 (CI: [1.8; 6.3]), 3.8 (CI: [1.9; 7.7]) and 4.2 (CI: [1.6; 11.1]), respectively, relative to nevirapine‐containing ART users (the reference group) (Table 2). There was little statistical evidence that the association between efavirenz and pregnancy changed over the length of time on the implant (interaction *p*‐value = 0.88). Among women who were using etonogestrel implants with efavirenz‐containing ART, HRs of pregnancy were 2.5 (CI: [1.0; 6.1]), 3.0 (CI: [1.5; 6.1]), 3.6 (CI: [1.6; 7.9]) and 4.3 (CI: [1.5; 12.9]), respectively, relative to nevirapine‐containing ART users (interaction *p*‐value = 0.69). HRs of pregnancy comparing women who were using levonorgestrel implants with efavirenz‐based ART to nevirapine‐containing ART users in the same periods were 12.2 (CI: [3.1; 48.2]), 8.4 (CI: [2.4; 29.5]), 5.8 (CI: [1.3; 25.3]) and 4.0 (CI: [0.6; 27.1]), respectively (interaction *p*‐value = 0.46). Likewise, the interactions between exposure and time were not statistically significant when comparing no ART use to nevirapine use (*p*‐values = 0.49, 0.44 and 0.75 for any implant, etonogestrel implant and levonorgestrel implant, respectively).

Results stratified by individual sampling phase can be found in Table S1. Among women included in the EMR sampling phase, HRs of pregnancy comparing efavirenz users to nevirapine users remained stable, albeit with a slight increase in HR over time among all implant users. Larger increases in relative hazards were observed over time among levonorgestrel users, but numbers of women at risk were very small at later time points. Similar findings existed for chart review and phone interview sampling phases, albeit with even smaller numbers of women and woman‐months at risk than in the EMR sampling phase. Estimates obtained from the complete case analysis are largely similar to those from the primary analyses using imputed datasets, suggesting minimal bias with our imputation approach (Table S1).

## 4 DISCUSSION

Among a cohort of WLHIV using ART and contraceptive implants in Kenya, an increased risk of a breakthrough pregnancy, or implant failure, was observed with efavirenz use relative to nevirapine use. However, we found no evidence that the relative hazard of pregnancy between these two ART use groups changed based on the length of time on the implant. Therefore, it does not appear that these implants are more fallible from drug–drug interactions with efavirenz at later time intervals of implant use. Thus, based on our data, we would not recommend shortening the duration of implant use or replacing the implants sooner when concomitantly used with efavirenz. However, to limit pregnancy risk given the demonstrated interactions between efavirenz and all implant types, the use of other first‐line ART regimens that have been shown to not interact with implants is preferable when possible.

Our finding is contrary to expectations, as efavirenz has been documented to reduce implant hormone concentrations by 40–82% in women concomitantly using both medications [11, 12, 13, 14]. Thus, logically, given the relative logarithmic decline in implant hormone concentrations after the first 6–12 months of use, one can easily hypothesize that a longer duration of concurrent use with efavirenz will further reduce implant hormone concentrations, allowing more frequent breakthrough pregnancies after those time points. On the other hand, Scarsi and colleagues documented three incident pregnancies due to implant failures in the first year of implant use among a cohort of 20 women they followed in a pharmacokinetic study of levonorgestrel implants and efavirenz use [13]. Our unpublished data from a similar pharmacokinetic study show a disproportionate number of events when endogenous progesterone (a surrogate marker for ovulation) was well above the accepted cut‐offs for ovulation within the first 6 months of either etonogestrel or levonorgestrel implant use while on efavirenz as compared to dolutegravir‐containing ART or no ART use [23]. It is also important to acknowledge that other mechanisms may play a role in how efavirenz reduces implant efficacy [24]. For example, recent pharmacogenomic studies have examined the association between CYP enzyme variants and progestin metabolism that may contribute to hormonal contraceptive failures. Limitations of our data should also be kept in mind here as we lack exact dates of implant placements and, rather, only have the first date implant use recorded in the EMR, which could have occurred after the implant placement visit. However, given that many of these facilities provide integrated HIV and family planning care, it is likely that clients underwent the implant placement at the same visit or soon before the visit where it is recorded.

A contraceptive failure, regardless of the method, can be quite distressing for a woman, her partner and her provider, and can lead to additional personal and health system costs [25, 26]. Failures with hormonal contraceptive methods, which are generally more effective than non‐hormonal methods (other than copper intrauterine devices or surgical methods), are particularly alarming. Ideally, all potential sources of contraceptive method failure, including drug–drug interactions, are explored well before the method is used at scale with its intended population. However, the phenomenon observed with implant failures with concomitant efavirenz use raises the issue of how little we understand how drug–drug interactions influence contraceptive efficacy. A part of that knowledge gap has to do with a limited understanding of the complex nature of how hormonal contraceptives prevent pregnancy in the first place [27]. Therefore, it is imperative that adequate investments in research be made to understand the fundamental mechanisms of contraceptive efficacy to give women, including those who are living with HIV, fully informed choices about their reproductive health.

The urgency to develop alternative solutions to the reduced effectiveness of contraceptive implants with efavirenz use has, fortunately, decreased. Now, the World Health Organization recommends dolutegravir‐containing ART as the first‐line ART choice for all adults living with HIV, including women of reproductive potential [28]. However, the initial rollout of dolutegravir‐containing ART was hampered significantly by a potential signal detection between dolutegravir exposure peri‐conception and neural tube defects among infants [29]. The policy and programme changes that ensued to remove dolutegravir as an option for WLHIV, unfortunately, have had lasting negative consequences. As recently as early 2021, a significant proportion of WLHIV of reproductive potential was still not transitioned to or started on dolutegravir‐containing ART [30]. Dolutegravir‐containing ART is anticipated to avoid any detrimental drug–drug interactions with contraceptive implants [23, 31]. For women not yet on dolutegravir, an undesirable trade‐off continues to exist between using efavirenz‐containing ART and facing reduced effectiveness of implants—the most effective contraceptive method available in most settings. Emerging data from real‐world use of newer ART regimens underscore those investments that prioritize research for women's reproductive health needs must occur far sooner and more upstream in the drug development and testing pipelines.

Though our study is the first to report on time to breakthrough pregnancy among efavirenz users and has a relatively large sample size, our work has several limitations. First, we lack data on exact implant insertion and removal dates, as this information was not explicitly noted in the EMR. Thus, it is possible that we have misclassified an incident pregnancy as a breakthrough pregnancy when the woman had the implant removed before her pregnancy. This may result in an overestimation of absolute breakthrough pregnancy rates; however, we do not believe any such misclassification is differential by ART regimen types, and our comparisons across the ART types should be valid. Second, we have little observation time of implant use past 48 months of use, and while it is possible that the risk of breakthrough pregnancy may increase significantly after 48 months, we do not see a signal of that phenomenon starting at 12 or 24 months (when progestin concentrations in the blood are already near their nadir), which indicates that such an association is ultimately highly unlikely. Third, we were unable to evaluate possible interactions of ART and tuberculosis treatment use on pregnancy outcomes due to few women reporting tuberculosis medication use. We also lacked data on potential confounders, such as sexual activity or condom use, that could bias our findings. Again, we do not believe these variables are likely to change over time or between ART regimen types. Fourth, many women included in this study did not contribute 48 months of follow‐up; patients who had shorter follow‐up periods may have been systemically different than those who had longer follow‐up periods in our study, which could have biased our results. Finally, given that this is a secondary analysis with few events (and women at risk) at longer follow‐up times, our power to assess changes in breakthrough pregnancies over time through time interaction models was limited.

## 5 CONCLUSIONS

In a retrospective longitudinal analysis of a large cohort of WLHIV who are using contraceptive implants in Kenya, we do not find any associations between breakthrough pregnancies, or implant failures, and duration of implant use among women concomitantly using efavirenz relative to nevirapine. Of the pregnancies we observed, no specific time interval of implant use appeared to signal a higher risk of breakthrough pregnancy, even when considering a specific implant type. Thus, we would not recommend shortening the duration of implant use or replacing the implants sooner when used concomitantly with efavirenz. Instead, we urge policymakers and programmes to promote dolutegravir‐containing ART use among WLHIV of reproductive potential, which does not appear to interact with implants to reduce effectiveness, especially among those already using implants and efavirenz.

## COMPETING INTERESTS

The authors declare that they have no competing interests.

## AUTHORS’ CONTRIBUTIONS

RCP, CRC and CTY conceptualized the primary research question and overall study design. RMS, ARM and RCP conceptualized this secondary analysis and study design, with RMS leading and ARM conducting the final analysis presented here. RCP, BJ, ARM, BM, CB, EAB, KW‐K, CRC and CTY led parts of or directly contributed to study implementation. RCP, ARM, CB and BJ contributed to data collection. GA, BES, RCP, CTY, RS and BM led or directly contributed to data analysis. RMS and RCP drafted the initial manuscript with major assistance from GA, and all authors contributed to results interpretation and manuscript revisions. All authors have read and approved the manuscript.

## FUNDING

This publication was made possible by support for AMPATH by U.S. President's Emergency Plan for AIDS Relief (PEPFAR) through joint support of the United States Agency for International Development (USAID; AID‐623‐A‐12‐0001). This publication was also made possible by support for FACES from PEPFAR through a cooperative agreement from the U.S. Centers for Disease Control and Prevention (CDC), Division of Global HIV/AIDS (PS001913). Research reported in this publication was supported by the National Institute of Allergy and Infectious Diseases (NIAID), Eunice Kennedy Shriver National Institute of Child Health & Human Development (NICHD), National Institute on Drug Abuse (NIDA), National Cancer Institute (NCI) and the National Institute of Mental Health (NIMH), in accordance with the regulatory requirements of the National Institutes of Health (NIH) for East Africa IeDEA Consortium (U01AI069911). Some study data were collected and managed using REDCap electronic data capture tools hosted at the Institute of Translational Health Sciences and supported by the National Center For Advancing Translational Sciences (NCATS) of the NIH (UL1 TR002319). Dr. Patel and some data collection were supported by NIAID of the NIH (K23AI120855). Drs. Amorim and Shepherd's effort on this work was supported by NIAID of the NIH (R01AI131771). Dr. Bernard was supported by the Clinical and Translational Science Award (CTSA) programme of the NCATS of the NIH under Award Numbers UL1 TR000448 and TL1 TR000449 and NIH Reproductive Epidemiology Training Grant number T32HD055172. The funders had no role in the study design, writing of this article or in the decision to submit this article for publication. All authors had full access to the data, take responsibility for the integrity and accuracy of the data, and had full independence from the funders.

## DISCLAIMER

The findings and conclusions in this paper are those of the authors and the contents are the sole responsibility of the authors and do not necessarily represent the official position or views of the U.S. Centers for Disease Control and Prevention, USAID, the National Institutes of Health, the United States Government or the Government of Kenya.

## Supporting information


**Figure S1**. Instantaneous hazard of breakthrough pregnancy among all sampled women, by implant type. Plots on the left correspond to the etonogestrel implant and plots on the right correspond to the levonorgestrel implant for data collected from electronic medical records (EMRs) (a), chart review (b) and telephone interview (c).

**Table S1** Risk of breakthrough pregnancy over time among women using contraceptive implant and nevirapine‐containing, efavirenz‐containing and no ART using Cox PH models, by sampling phase.

## Data Availability

The data that support the findings of this study are available on request from the corresponding author upon reasonable request. The data are not publicly available due to privacy or ethical restrictions.
