# Supplementary figures and images for: Contraceptive implant use duration is not associated with breakthrough pregnancy among women living with HIV and using efavirenz: a retrospective, longitudinal analysis
Source: J Int AIDS Soc. 2022 Sep 8;25(9):e26001. doi: 10.1002/jia2.26001 (PMC9454412; doi:10.1002/jia2.26001)

A) Electronic medical record (EMR)

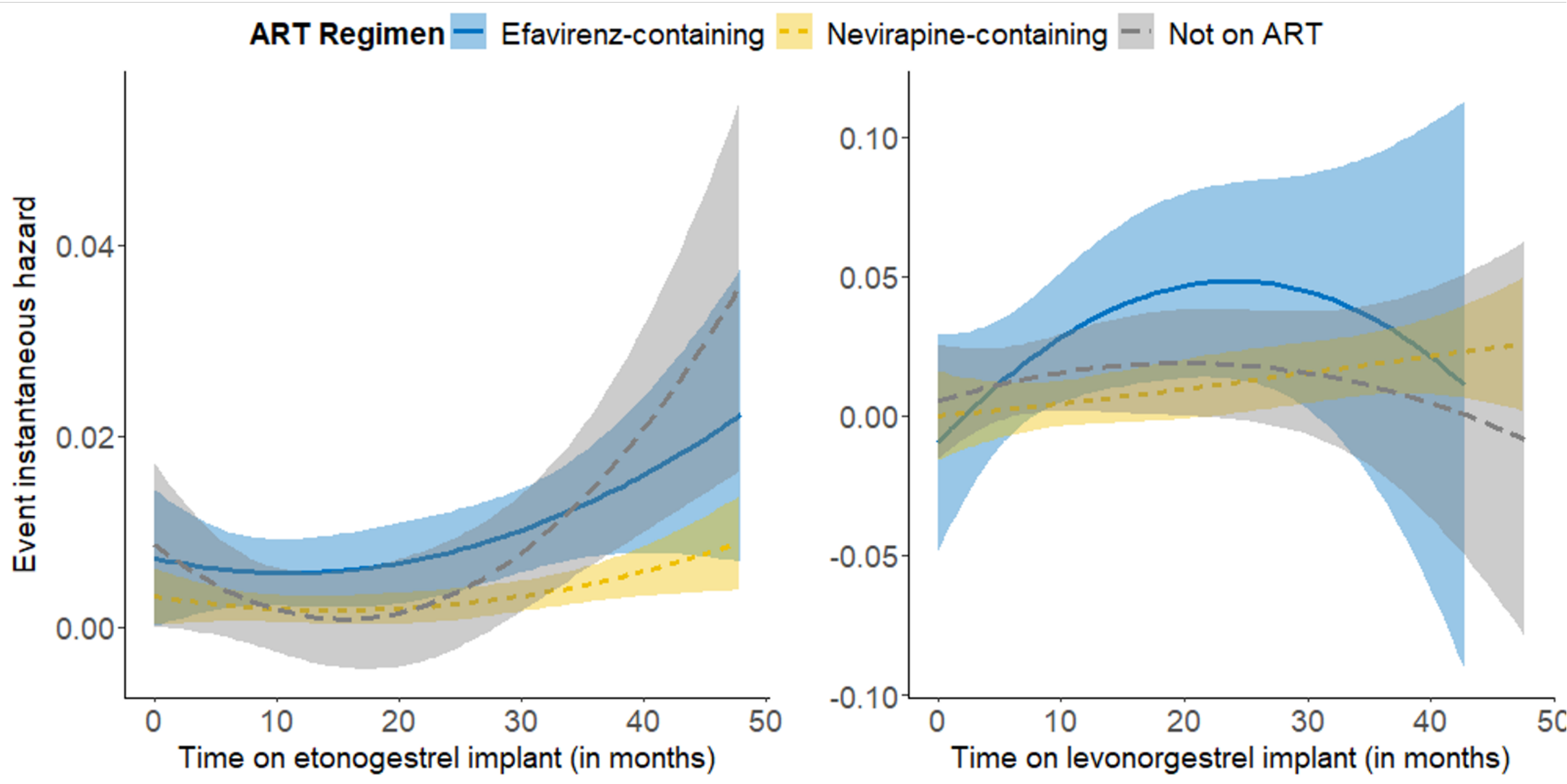

B) Chart review

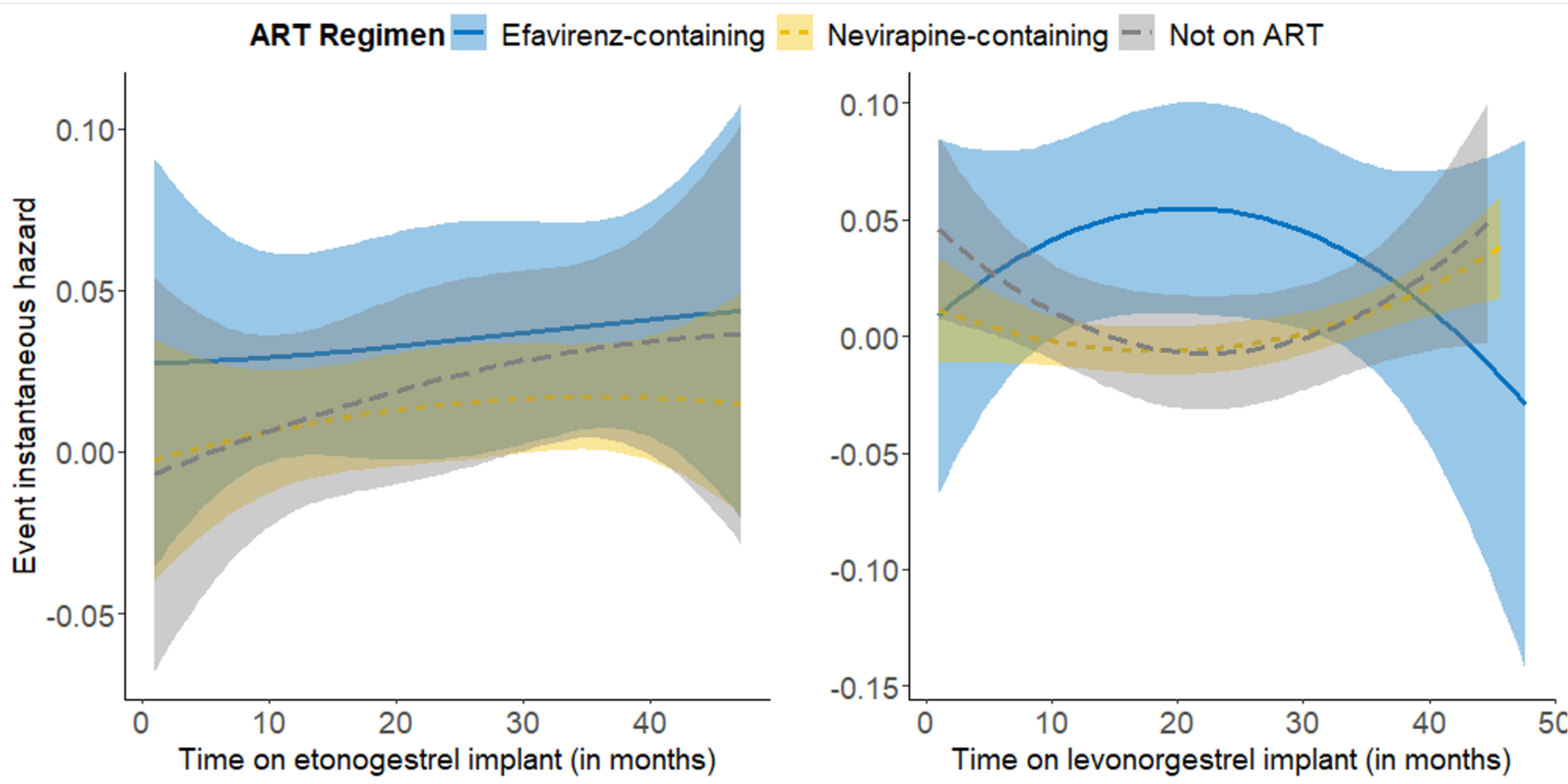

B) Telephone interview

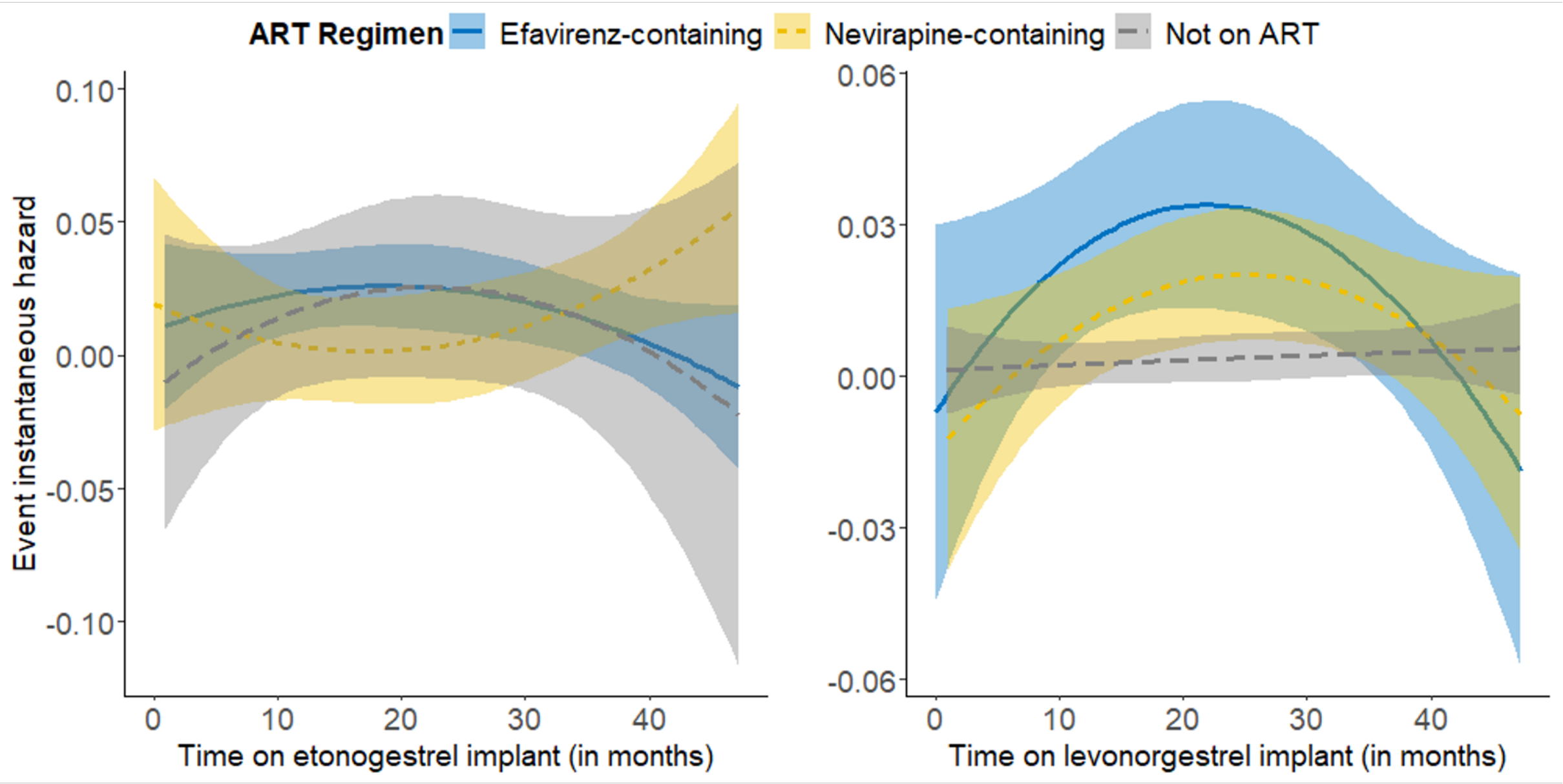

Supplement: Supplementary file 1 — Figure S1. Instantaneous hazard of breakthrough pregnancy among all sampled women, by implant type. Plots on the left correspond to the etonogestrel implant and plots on the right correspond to the levonorgestrel implant for data collected from electronic medical records (EMRs) (a), chart review (b) and telephone interview (c). [file JIA2-25-e26001-s001.pdf]
