# Supplementary material for: Contraceptive implant use duration is not associated with breakthrough pregnancy among women living with HIV and using efavirenz: a retrospective, longitudinal analysis
Source: J Int AIDS Soc. 2022 Sep 8;25(9):e26001. doi: 10.1002/jia2.26001 (PMC9454412; doi:10.1002/jia2.26001)
Supplement: Supplementary file 2 — Table S1 Risk of breakthrough pregnancy over time among women using contraceptive implant and nevirapine‐containing, efavirenz‐containing and no ART using Cox PH models, by sampling phase. [file JIA2-25-e26001-s002.docx]

**Supplemental Table 1. Risk of breakthrough pregnancy over time among women using contraceptive implant and nevirapine-containing, efavirenz-containing and no ART using Cox PH models, by sampling phase.**

|  |  | **Efavirenz versus nevirapine** | | | |  | | **No ART versus nevirapine** | | | |
| --- | --- | --- | --- | --- | --- | --- | --- | --- | --- | --- | --- |
|  | **EMR - Complete case**  **(1^ST^ PHASE)** ^§^ | | **EMR**  **(1^ST^ PHASE)** | **CHART REVIEW**  **(2^ND^ PHASE)** | **TELEPHONE INTERVIEW**  **(3^RD^ PHASE)** | | **EMR - Complete case**  **(1^ST^ PHASE)** ^§^ | | **EMR**  **(1^ST^ PHASE)** | **CHART REVIEW**  **(2^ND^ PHASE)** | **TELEPHONE INTERVIEW**  **(3^RD^ PHASE)** |
| **Months since implant insertion** | **aHR**  **(95% CI)**^†^ | | **aHR**  **(95% CI)**^†^ | **aHR**  **(95% CI)**^‡^ | **aHR**  **(95% CI)**^‡^ | | **aHR**  **(95% CI)**^†^ | | **aHR**  **(95% CI)**^†^ | **aHR**  **(95% CI)**^‡^ | **aHR**  **(95% CI)**^‡^ |
| **Any Implant** |  | |  |  |  | |  | |  |  |  |
| **Month 6** | 2.1 (1.6-2.7) | | 2.1 (1.7-2.6) | 3.4 (2.1-5.8) | 2.7 (1.2-6.0) | | 1.3 (0.9-1.7) | | 1.3 (1.0-1.7) | 1.9 (0.9-3.7) | 1.3 (0.5-3.4) |
| **Month 12** | 2.0 (1.6-2.6) | | 2.1 (1.7-2.6) | 2.9 (1.9-4.3) | 2.7 (1.4-5.5) | | 1.3 (1.0-1.7) | | 1.3 (1.0-1.7) | 1.6 (0.9-2.6) | 1.4 (0.6-3.1) |
| **Month 18** | 2.0 (1.4-2.6) | | 2.1 (1.6-2.8) | 2.4 (1.7-3.3) | 2.8 (1.6-5.2) | | 1.3 (0.9-1.8) | | 1.3 (1.0-1.8) | 1.3 (0.8-2.0) | 1.5 (0.7-2.9) |
| **Month 24** | 1.9 (1.3-2.8) | | 2.1 (1.4-3.0) | 2.0 (1.4-2.8) | 2.9 (1.7-5.1) | | 1.3 (0.9-2.1) | | 1.3 (0.9-2.0) | 1.1 (0.6-1.8) | 1.5 (0.8-2.9) |
| **Month 30** | 1.8 (1.1-3.1) | | 2.1 (1.3-3.4) | 1.7 (1.1-2.5) | 3.0 (1.7-5.3) | | 1.3 (0.8-2.4) | | 1.3 (0.8-2.3) | 0.9 (0.5-1.8) | 1.6 (0.9-3.1) |
| **Month 36** | 1.8 (0.9-3.4) | | 2.1 (1.2-3.8) | 1.4 (0.8-2.3) | 3.2 (1.7-5.9) | | 1.4 (0.7-2.8) | | 1.3 (0.7-2.6) | 0.8 (0.3-1.9) | 1.7 (0.8-3.6) |
| **Month 42** | 1.7 (0.8-3.7) | | 2.1 (1.0-4.3) | 1.2 (0.6-2.2) | 3.3 (1.6-6.9) | | 1.4 (0.6-3.2) | | 1.3 (0.6-3.0) | 0.6 (0.2-2.0) | 1.8 (0.8-4.3) |
| **Month 48** | 1.6 (0.7-4.2) | | 2.1 (0.9-4.9) | 1.0 (0.4-2.2) | 3.4 (1.4-8.2) | | 1.4 (0.5-3.8) | | 1.3 (0.5-3.5) | 0.5 (0.1-2.1) | 1.9 (0.7-5.4) |
| **Etonogestrel Implant** |  | |  |  |  | |  | |  |  |  |
| **Month 6** | 2.3 (1.6-3.4) | | 2.0 (1.4-2.7) | 3.1 (0.9-10.4) | 2.2 (0.8-5.8) | | 1.4 (0.9-2.0) | | 1.3 (0.9-1.8) | 1.5 (0.3-7.1) | 1.2 (0.4-3.4) |
| **Month 12** | 2.3 (1.6-3.2) | | 2.1 (1.6-2.8) | 2.7 (1.0-6.9) | 2.3 (1.0-5.4) | | 1.3 (0.9-1.8) | | 1.2 (0.9-1.7) | 1.4 (0.4-4.6) | 1.3 (0.5-3.1) |
| **Month 18** | 2.3 (1.6-3.3) | | 2.2 (1.6-3.1) | 2.3 (1.1-4.8) | 2.5 (1.2-5.1) | | 1.2 (0.8-1.8) | | 1.2 (0.8-1.8) | 1.2 (0.5-3.3) | 1.4 (0.6-2.9) |
| **Month 24** | 2.2 (1.4-3.7) | | 2.3 (1.5-3.6) | 2.0 (1.0-3.7) | 2.7 (1.4-5.2) | | 1.1 (0.6-1.9) | | 1.1 (0.6-1.9) | 1.1 (0.4-2.7) | 1.4 (0.7-3.0) |
| **Month 30** | 2.2 (1.2-4.2) | | 2.4 (1.4-4.3) | 1.7 (0.8-3.4) | 2.9 (1.5-5.7) | | 1.0 (0.5-2.1) | | 1.1 (0.5-2.2) | 1.0 (0.4-2.8) | 1.5 (0.7-3.3) |
| **Month 36** | 2.2 (1.0-4.9) | | 2.5 (1.2-5.2) | 1.4 (0.6-3.5) | 3.2 (1.5-6.6) | | 0.9 (0.4-2.4) | | 1.0 (0.4-2.5) | 0.9 (0.2-3.2) | 1.6 (0.7-3.9) |
| **Month 42** | 2.2 (0.8-5.7) | | 2.7 (1.1-6.3) | 1.2 (0.4-3.9) | 3.4 (1.4-8.1) | | 0.9 (0.3-2.7) | | 0.9 (0.3-2.8) | 0.8 (0.2-4.0) | 1.7 (0.6-4.9) |
| **Month 48** | 2.2 (0.7-6.7) | | 2.8 (1.0-7.7) | 1.0 (0.2-4.4) | 3.7 (1.3-10.2) | | 0.8 (0.2-3.0) | | 0.9 (0.3-3.2) | 0.7 (0.1-5.2) | 1.9 (0.5-6.3) |
| **Levonorgestrel implant** |  | |  |  |  | |  | |  |  |  |
| **Month 6** | 3.1 (0.8-11.5) | | 2.7 (0.9-7.8) | 2.3 (0.7-7.7) | 13.6 (1.9-98.7) | | 1.5 (0.4-5.0) | | 1.3 (0.5-3.6) | 1.0 (0.2-6.0) | 3.8 (0.3-44.9) |
| **Month 12** | 3.7 (1.3-10.5) | | 3.3 (1.4-7.7) | 2.1 (0.8-5.3) | 10.3 (1.9-54.9) | | 1.6 (0.6-4.1) | | 1.3 (0.6-3.0) | 1.0 (0.3-3.7) | 3.4 (0.4-26.9) |
| **Month 18** | 4.5 (1.8-11.1) | | 4.1 (1.9-8.8) | 1.9 (0.9-3.9) | 7.8 (1.9-32.3) | | 1.6 (0.7-3.7) | | 1.4 (0.6-3.0) | 0.9 (0.3-2.5) | 3.1 (0.6-16.9) |
| **Month 24** | 5.4 (2.0-14.5) | | 5.1 (2.1-12.3) | 1.7 (0.9-3.1) | 5.9 (1.7-20.7) | | 1.7 (0.7-4.1) | | 1.4 (0.6-3.4) | 0.9 (0.4-2.0) | 2.8 (0.7-11.6) |
| **Month 30** | 6.5 (1.9-22.4) | | 6.4 (2.1-19.7) | 1.5 (0.7-3.0) | 4.4 (1.3-15.1) | | 1.8 (0.6-5.3) | | 1.4 (0.5-4.4) | 0.8 (0.3-2.2) | 2.5 (0.7-9.2) |
| **Month 36** | 7.9 (1.6-38.0) | | 7.9 (1.9-33.7) | 1.3 (0.5-3.2) | 3.3 (0.9-12.5) | | 1.9 (0.5-7.4) | | 1.5 (0.4-6.0) | 0.7 (0.2-2.9) | 2.2 (0.6-8.7) |
| **Month 42** | 9.5 (1.3-67.4) | | 9.8 (1.6-59.4) | 1.2 (0.4-3.7) | 2.5 (0.5-11.6) | | 2.0 (0.4-10.9) | | 1.5 (0.3-8.5) | 0.7 (0.1-4.1) | 2.0 (0.4-9.8) |
| **Month 48** | 11.4 (1.1-122.1) | | 12.2 (1.4-106.7) | 1.1 (0.3-4.5) | 1.9 (0.3-11.6) | | 2.1 (0.3-16.5) | | 1.5 (0.2-12.1) | 0.6 (0.1-6.2) | 1.8 (0.3-12.2) |
| **Unknown implant** |  | |  |  |  | |  | |  |  |  |
| **Month 6** | 2.2 (1.4-3.5) | | 2.0 (1.4-2.9) | 4.0 (2.0-8.0) | - | | 1.1 (0.6-2.1) | | 1.4 (0.8-2.2) | 2.4 (1.0-5.8) | - |
| **Month 12** | 2.1 (1.1-4.2) | | 2.0 (1.1-3.6) | 3.3 (1.9-5.5) | - | | 1.7 (0.8-3.8) | | 1.7 (0.9-3.5) | 1.8 (0.9-3.5) | - |
| **Month 18** | 2.0 (0.7-5.9) | | 2.0 (0.8-5.0) | 2.6 (1.7-4.2) | - | | 2.7 (0.9-8.7) | | 2.2 (0.7-6.7) | 1.3 (0.6-2.9) | - |
| **Month 24** | 1.9 (0.4-8.5) | | 1.9 (0.5-7.1) | 2.1 (1.2-3.7) | - | | 4.4 (0.9-21.7) | | 2.9 (0.6-13.2) | 1.0 (0.3-3.0) | - |
| **Month 30** | 1.8 (0.3-12.4) | | 1.9 (0.4-10.3) | 1.7 (0.8-3.6) | - | | 6.9 (0.9-55.6) | | 3.7 (0.5-26.6) | 0.7 (0.2-3.3) | - |
| **Month 36** | 1.7 (0.2-18.2) | | 1.9 (0.2-15.1) | 1.4 (0.5-3.6) | - | | 11.0 (0.8-143.9) | | 4.7 (0.4-54.0) | 0.5 (0.1-3.9) | - |
| **Month 42** | 1.6 (0.1-26.8) | | 1.9 (0.2-22.0) | 1.1 (0.3-3.7) | - | | 17.5 (0.8-375.2) | | 6.0 (0.3-110.0) | 0.4 (0.0-4.6) | - |
| **Month 48** | 1.5 (0.1-39.5) | | 1.9 (0.1-32.2) | 0.9 (0.2-3.8) | - | | 27.7 (0.8-982.0) | | 7.7 (0.3-224.4) | 0.3 (0.0-5.4) | - |
| aHR: adjusted hazard ratio; ART: antiretroviral therapy; CI: confidence interval; EMR: electronic medical record;  - Insufficient sample size to estimate hazard ratios.  ^†^Calculated using proportional Cox regression models, adjusting for age, body mass index (log-transformed), any number of living children, program, and an interaction of ART regimen with time, using robust standard errors.  ^‡^Calculated using proportional Cox regression models adjusting for age, program, and an interaction of ART regimen with time, using robust standard errors.  ^§^Complete-case analysis by excluding all observations with missing information for adjustment variables | | | | | | | | | | | |
